# Renal mineralocorticoid receptor expression is reduced in lipoatrophy

**DOI:** 10.1002/2211-5463.12579

**Published:** 2019-01-18

**Authors:** Barbara Toffoli, Stella Bernardi, Carine Winkler, Coralie Carrascosa, Federica Gilardi, Béatrice Desvergne

**Affiliations:** ^1^ Center for Integrative Genomics Faculty of Biology and Medicine University of Lausanne Switzerland; ^2^ Department of Medical Sciences University of Trieste Italy

**Keywords:** kidney, lipoatrophy, mineralocorticoid receptor, obesity

## Abstract

Obesity is a condition characterized by adipose tissue hypertrophy; it is estimated that the obesity epidemic accounted for 4 million deaths in 2015 and that 70% of these were due to cardiovascular disease (CVD). One of the mechanisms linking obesity to CVD is the ability of adipose tissue to secrete circulating factors. We hypothesized that adipose tissue and its secretory products may influence mineralocorticoid receptor (MR) expression. Here, we showed that expression of MR and its downstream targets (*Cnksr3*,* Scnn1b*, and *Sgk1*) were significantly reduced in the kidneys of peroxisome proliferator‐activated receptor‐γ null (*Pparg* ^*Δ/Δ*^) and A‐ZIP/F‐1 (AZIP^tg/+^) lipoatrophic mice with respect to their controls. Intriguingly, MR expression was also found to be significantly reduced in the kidneys of genetically obese ob/ob mice. Our data suggest that adipose tissue contributes to the regulation of MR expression. Given that leptin deficiency seems to be the major feature shared by *Pparg* ^*Δ/Δ*^, AZIP^tg/+^, and ob/ob mice, we speculate that adipose tissue modulates MR expression through the leptin system.

**Abbreviations**
CVD: cardiovascular disease
ENaC: epithelial Na channel
MR: mineralocorticoid receptor
Nr3c2: nuclear receptor subfamily 3 group C member 2
PPARγ: peroxisome proliferator‐activated receptor‐γ
RAAS: renin–angiotensin–aldosterone system
Scnn1b: sodium channel epithelial 1 beta subunit
Sgk1: serum/glucocorticoid regulated kinase 1

Obesity is a condition characterized by adipose tissue hypertrophy, whose prevalence is increasing worldwide. It is estimated that the obesity epidemic accounted for 4 million deaths in 2015, and that 70% of them were due to cardiovascular disease (CVD) 1. One of the mechanisms linking obesity to CVD is the ability of the adipose tissue to secrete circulating factors leading to organ damage. In obesity, for example, there is an activation of adipose renin–angiotensin–aldosterone system (RAAS), with subsequent activation of systemic RAAS, ultimately promoting CVD 2, 3. Consistent with this, obese patients exhibit higher levels of aldosterone, which have been associated with a greater risk for cardiometabolic disease 3.

Aldosterone is a mineralocorticoid hormone that is primarily synthetized in the adrenal cortex. Its biological actions are mediated by the mineralocorticoid receptors (MR), which are located in epithelial and nonepithelial tissues. After binding to MR, aldosterone does not only lead to sodium reabsorption in the kidney, whereby it regulates blood pressure, but it also promotes widespread organ damage 4.

Mouse strains with lipoatrophy, which is a generalized loss of adipose tissue, have been used to understand the role of white adipose tissue in health and disease states. These strains include the peroxisome proliferator‐activated receptor‐γ (PPARγ) null 5, 6 and the A‐ZIP/F‐1 mice 7. In PPARγ null mice, lipoatrophy is due to the lack of PPARγ, which is essential for adipose tissue development 5. In A‐ZIP/F‐1 mice, the adipose‐selective expression of a dominant negative protein, A‐ZIP/F, impairs normal adipocyte growth and differentiation 7.

We hypothesized that adipose tissue and its secretory products could influence MR regulation. To test this hypothesis, we evaluated the renal expression of MR in mouse models of lipoatrophy and compared it with that of mouse models of obesity.

## Materials and methods

### Animal models

Female PPARγ null (*Pparg* ^*Δ/Δ*^) mice were generated in our laboratory as previously described 5, 6. Littermates with a mixed C57BL/6J × 129 genetic background and two functional *Pparg* alleles were used as controls (CTL). Female A‐ZIP/F‐1 mice (AZIP^tg/+^) and their wild‐type controls on a FVB/N background (FVB/N), which were a kind gift from C. Vinson, were generated as previously reported 7. Female B6.V‐*Lep* ^*ob*^/J (ob/ob) mice and their controls (C57BL/6J) were purchased from Charles River (Saint Germain Nuelles, France).

Mice were followed for different time‐periods and sacrificed by CO_2_ inhalation. Kidneys were homogenized for protein extraction or snap frozen for RNA analysis. Skin samples were snap frozen in 1 mL of TRI‐reagent/sample (Thermo Fisher Scientific, Waltham, MA, USA). Leptin was measured by ELISA (R&D, Minneapolis, MN, USA; MOB00); creatinine, glucose, and plasma aldosterone were measured at the Nephrology Service (CHUV, Switzerland). Animal care and treatments were carried out in compliance with specific European laws (86/609/EEC). This study was approved by the Commission for Animal Experimentation of the Cantonal Veterinary Services (Canton of Vaud).

### Quantitative real‐time RT‐PCR

Total RNA from kidney was isolated with TRI‐Reagent and RNeasy Mini Kit (Qiagen, Hilden, Germany). Gene expression of *Nr3c2* and downstream mediators (*Cnksr3, Scnn1b, Sgk1*) was analyzed by real‐time quantitative PCR (FastStart Universal SYBR Green Master; Roche, Pleasanton, CA, USA) in a Stratagene MX3005P Detection System (Agilent Technologies, Santa Clara, CA, USA). *Rps9* was used as the housekeeping gene. Primer sequences are available in Table 1.

**Table 1:** List of primers

Gene	Primer pair
Mouse	
*Rps9*	(F) 5′‐GACCAGGAGCTAAAGTTGATTGGA‐3′
	(R) 5′‐TCTTGGCCAGGGTAAACTTGA‐3′
*Nr3c2*	(F) 5′‐TCCTTTCCGCCTGTCAATG‐3′
	(R) 5′‐GAGGATCCAGTAGAAACACTTCG‐3′
*Cnksr3*	(F) 5′‐GACTCCTGTCGATTGCCTAG‐3′
	(R) 5′‐TCTCCCGCTCAAACTTGTG‐3′
*Scnn1b*	(F) 5′‐CCCTGATCGCATAATCCTAGC‐3′
	(R) 5′‐ATGCCCCAGTTGAAGATGTAG‐3′
*Sgk1*	(F) 5′‐CTTATGAACGCTAACCCCTCTC‐3′
	(R) 5′‐GAACCTTTCCAAAACTGCCC‐3′

### Western blot

Fresh kidney samples were manually homogenized in ice‐cold TEN buffer containing protease inhibitors. After centrifugation, cells were resuspended in buffer A (10 mm HEPES pH 7.9, 10 mm KCl, 0.1 mm EGTA pH8, 0.1 mm EDTA, 1 mm DTT) and subsequently lysed by the addition of 10% NP‐40. The homogenate was centrifuged, and the supernatant containing the cytoplasmic fraction was collected and stored at −80 °C. The pellet was then resuspended in nuclear extraction buffer, put on ice, and mixed periodically for 20 min. After centrifugation, the supernatant containing the nuclear fraction was stored at −80 °C.

Protein quantification was performed by BCA protein assay kit (Thermo Scientific). Cytosolic and nuclear fractions were subjected to SDS/PAGE and blotted onto nitrocellulose filters. After blocking, the membranes were incubated with primary antibodies for MR (MRN 2B7, a kind gift from C. Gomez‐Sanchez), followed by peroxidase‐conjugated goat anti‐mouse secondary antibodies. Immunoreactivity was detected using the Supersignal West Pico chemiluminescent substrate (Thermo Scientific). β‐Actin (Sigma‐Aldrich, St. Louis, MO, USA), GAPDH (Cell Signaling Technology, Danvers, MA, USA), and U2AF (Sigma‐Aldrich) were used as loading controls.

### Statistical analysis

Values, expressed as mean ± SEM, were analyzed using prism 5.0 (GraphPad Software, San Diego, CA, USA). Student's *t*‐test was used to assess statistical significance. A *P* value < 0.05 was considered statistically significant.

## Results

### MR expression is significantly reduced in the kidneys of *Pparg* ^*Δ/Δ*^ and AZIP^tg/+^ mice

**Figure 1:**
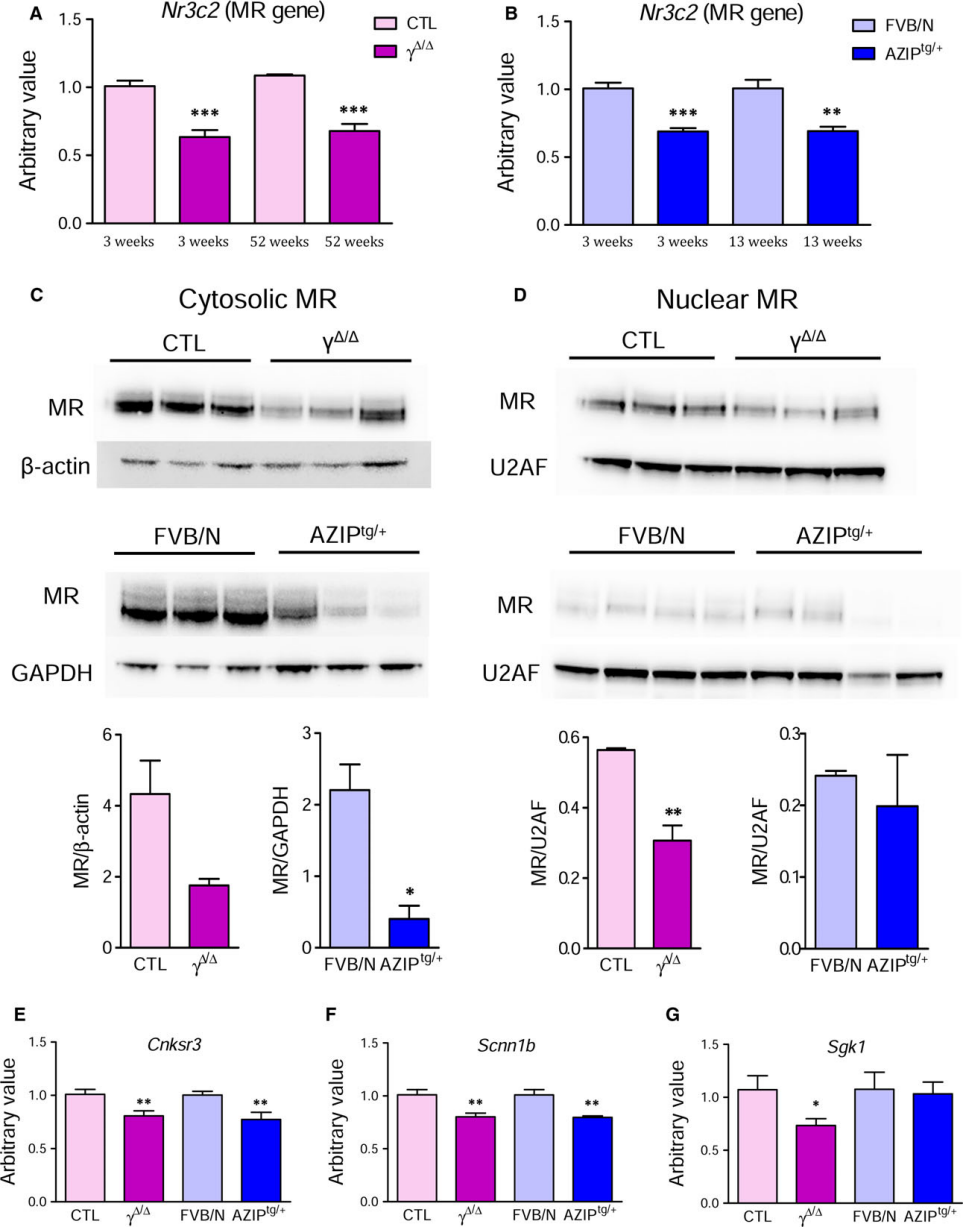
MR is downregulated in kidneys of lipoatrophic mice. (A) Renal *Nr3c2* (MR gene) expression evaluated by RT‐qPCR in CTL and *Pparg* ^*Δ/Δ*^ (γ^*Δ/Δ*^) mice at 3 and 52 weeks of age (*n* = 5–11). (B) Renal *Nr3c2* expression in FVB/N and AZIP^tg/+^ animals at 3 and 13 weeks of age (*n* = 4–8). Results in *Pparg* ^*Δ/Δ*^ and AZIP^tg/+^ mice are expressed as fold modulation with respect to their related littermate levels, which were arbitrarily set to 1. Data show mean ± SEM. **P <* *0.01, ***P <* *0.0001 *Pparg* ^*Δ/Δ*^ and AZIP^tg/+^ *vs*. CTL or FVB/N, respectively (Student's *t*‐test). (C) Representative blots and densitometric analysis of MR protein in the renal cytosolic fraction of 3‐week‐old CTL, *Pparg* ^*Δ/Δ*^ (γ^*Δ/Δ*^), FVB/N, and AZIP^tg/+^ mice. Data, normalized to β‐actin or GAPDH, are expressed as mean ± SEM (*n* = 3). (D) Nuclear MR protein expression in kidneys of the same groups of mice. Data, normalized to U2AF, are expressed as mean ± SEM (*n *= 3–4). *P <* *0.05, **P <* *0.01 *Pparg* ^*Δ/Δ*^ and AZIP^tg/+^ *vs*. relative control animals (Student's *t*‐test). (E) Renal expression of *Cnksr3*, (F) *Scnn1b*, and (G) *Sgk1* in CTL, *Pparg* ^*Δ/Δ*^ (γ^*Δ/Δ*^), FVB/N and AZIP^tg/+^ mice at 3 weeks of age (*n* = 6–11). Data show mean ± SEM. *P <* *0.05, **P <* *0.01 *Pparg* ^*Δ/Δ*^ and AZIP^tg/+^ *vs*. CTL or FVB/N, respectively (Student's *t*‐test).

**Figure 2:**
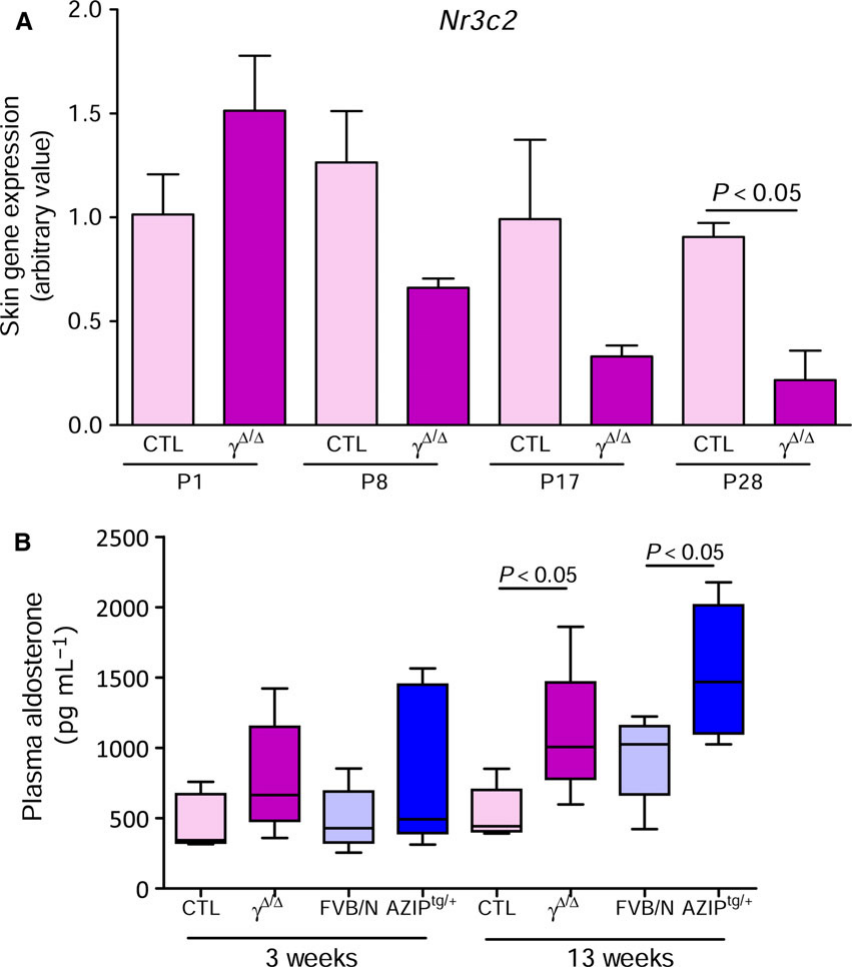
Skin MR expression and aldosterone levels in lipoatrophic mice. (A) Skin *Nr3c2* expression evaluated by RT‐qPCR in CTL and *Pparg* ^*Δ/Δ*^ (γ^*Δ/Δ*^) mice at different time points (P1, P8, P17, and P28 days) after birth (*n* = 3–5). Data show mean ± SEM. Student's *t*‐test was used to assess statistical significance. (B) Box plots represent plasma aldosterone evaluated at 3 and 13 weeks of age in CTL, *Pparg* ^*Δ/Δ*^ (γ^*Δ/Δ*^), FVB/N, and AZIP^tg/+^ female mice (*n* = 5). Student's *t*‐test was used to assess statistical significance.

The general characteristics of the animal studied are reported in Table 2. Lipoatrophy was associated with body weight and leptin reduction, as well as kidney hypertrophy 5, 6. Interestingly, 3‐week‐old *Pparg* ^*Δ/Δ*^ and AZIP^tg/+^ mice exhibited a significant downregulation of MR gene (*Nr3c2*) expression in their kidneys, which was also observed in older animals (Fig. 1A,B). Similar results were found in the skin, suggesting that MR gene downregulation was tissue‐independent (Fig. 2A). The reduction of MR gene expression was associated with a significant reduction of cytosolic and nuclear MR protein levels, as assessed by western blot analysis, in the kidneys of 3‐week‐old lipoatrophic mice with respect to their controls (Fig. 1C,D).

**Table 2:** General characteristics of the mice studied

Parameters	CTL	*Pparg* ^*Δ/Δ*^	FVB/N	AZIP^tg/+^	C57BL/6J	ob/ob
Age (weeks)	3	3	3	3	8	8
Body weight (g)	11.6 ± 0.3	7.8 ± 0.2‡	12.6 ± 0.5	8.7 ± 0.7‡	20.6 ± 0.7	39.7 ± 0.6‡
Index of renal hypertrophy [(g/g) × 100]	1.26 ± 0.02	1.62 ± 0.04‡	1.18 ± 0.03	1.37 ± 0.03†	0.75 ± 0.01	1.23 ± 0.02‡
Plasma creatinine (μmol/L)	10.3 ± 0.2	10.9 ± 0.6	13.0 ± 0.4	14.1 ± 1.1	15.9 ± 1.8	21.2 ± 7.1
Glycemia (mmol/L)	10.1 ± 0.2	16.4 ± 1.5‡	12.2 ± 0.3	16.3 ± 0.9†	9.0 ± 0.1	14.5 ± 1.7†
Leptin (pg/mL)	1484.0 ± 491.0	121.4 ± 71.1*	1808.2 ± 344.3	205.3 ± 65.1†	1250.0 ± 629.6	Undetectable
Aldosterone (pg/mL)	467.4 ± 87.5	785.5 ± 179.5	492.6 ± 100.2	835.5 ± 255.4	347.2 ± 25.5	382.8 ± 30

Data are presented as mean ± SEM (*n *= 4–11). *P <* *0.05, ^†^ *P *<* *0.01, ^‡^ *P *<* *0.0001 *Pparg* ^*Δ/Δ*^ *vs*. CTL, AZIP^tg/+^ *vs*. FVB/N, or ob/ob *vs*. C57BL/6J (Student's *t*‐test).

### Downstream targets of MR are significantly reduced in the kidneys of *Pparg* ^*Δ/Δ*^ and AZIP^tg/+^ mice

Classically, when aldosterone binds to MR, it increases renal sodium reabsorption by upregulating the epithelial Na channel (ENaC) and the sodium/potassium ATPase (Na‐K‐ATPase) in the collecting duct system 3. *Cnksr3*,* Scnn1b*, and *Sgk1* are involved in this aldosterone‐mediated ENaC modulation through MR activation 8, 9. Consistent with the MR reduction, *Cnksr3* and *Scnn1b* were downregulated in both *Pparg* ^*Δ/Δ*^ and AZIP^tg/+^ mice, and Sgk1 was reduced in *Pparg* ^*Δ/Δ*^ mice (Fig. 1E–G). Conversely, both lipoatrophic models exhibited a progressive increase of aldosterone plasma levels, possibly due to the decrease of its specific receptor (Fig. 2B).

### MR expression is significantly decreased in the kidneys of ob/ob mice

**Figure 3:**
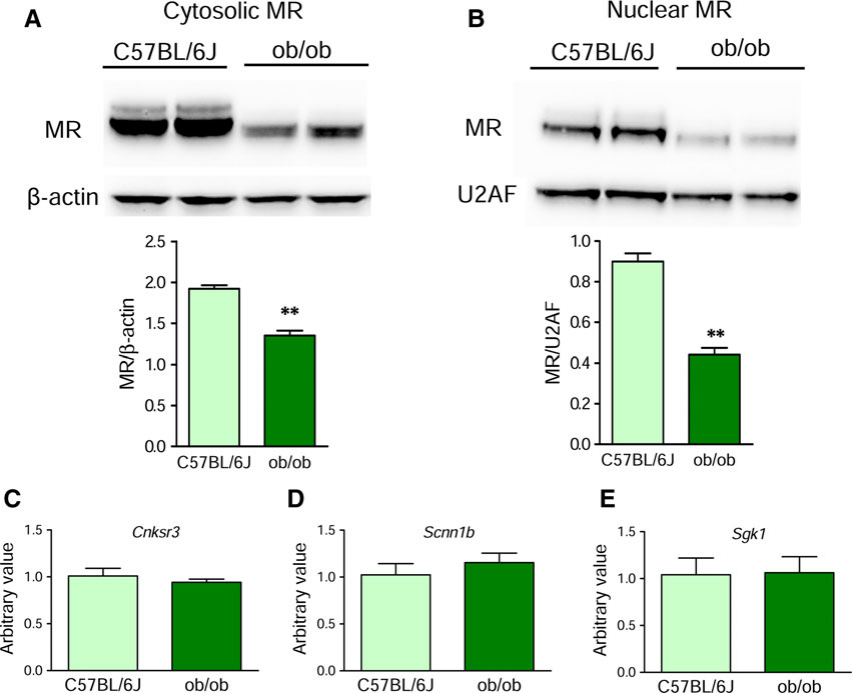
MR is downregulated in kidneys of ob/ob mice. (A) Representative blots and densitometric analysis of MR protein in the renal cytosolic fraction of 8‐week‐old ob/ob and control (C57BL/6J) mice. Data are normalized to β‐actin and expressed as mean ± SEM (*n* = 4). (B) Nuclear MR protein expression in kidneys of the same groups of mice. Data are normalized to U2AF and expressed as mean ± SEM (*n* = 4). **P <* *0.01 ob/ob *vs*. control animals (Student's *t*‐test). (C) Renal expression of *Cnksr3*, (D) *Scnn1b*, and (E) *Sgk1* in C57BL/6J and ob/ob mice at 8 weeks of age. Data show mean ± SEM (*n* = 4–5) (Student's *t*‐test).

To evaluate whether renal MR reduction in lipoatrophic mice was due to the absence of fat, we measured MR expression in the kidneys of ob/ob mice, a model of extreme obesity, due to a mutation of the gene encoding for leptin 10, 11. The general characteristics of these mice are reported in Table 2. Interestingly, we found that ob/ob mice displayed a significant reduction of both cytosolic and nuclear MR expression (Fig. 3A,B). Contrary to lipoatrophic mice, this was not associated with significant changes in aldosterone levels and/or the gene expression of *Cnksr3*,* Scnn1b*, and *Sgk1* (Fig. 3C–E).

## Discussion

This study shows that renal MR is significantly reduced not only in lipoatrophic mice but also in a mouse model of extreme obesity. Notwithstanding the presence of minor interstrain differences, our data suggest that the adipose tissue contributes to the regulation of renal MR expression, which seems related to adipose tissue function, rather than adipose tissue mass per se. Typically, adipose tissue dysfunction includes not only visceral (ectopic) fat accumulation (as seen in lipoatrophy), but also changes in its composition as well as in mRNA and protein expression patterns (as seen in ob/ob mice) 12.

In healthy conditions, adipocytes are metabolically active cells that secrete a wide variety of hormones and adipokines, such as leptin, which regulates several physiological functions 13 by binding to its specific receptors in different tissues, including kidney and skin 14, 15. Interestingly, leptin deficiency has been associated with insulin resistance, diabetes, and organ damage in humans and animals 5, 13. In addition, although transgenic overexpression of leptin and fat transplantation rescued the metabolic disorders in lipoatrophic AZIP^tg/+^ mice 16, 17, the transplantation of adipose tissue from ob/ob mice was unable to reverse AZIP^tg/+^ diabetes phenotype, thus underlying the fundamental role of leptin and its signaling in maintaining body homeostasis 18.

Adipocytes produce 19 and regulate aldosterone release as well. In particular, Ehrhart‐Bornstein observed that adipocytes isolated from healthy subjects secreted potent mineralocorticoid‐releasing factors, with a major effect on aldosterone release 20. It has been shown that leptin is one of these mineralocorticoid‐releasing factors, as it was able to directly regulate aldosterone secretion from the adrenal cortex, independent of angiotensin, and the sympathetic nervous system 21. Given that leptin deficiency seems the major feature that *Pparg* ^*Δ/Δ*^, AZIP^tg/+^, and ob/ob mice have in common, we speculate that adipose tissue modulates renal MR expression through leptin or a leptin‐regulated factor. This is consistent with the observation that in high‐fat diet‐induced obesity, which is associated with an increase of circulating leptin levels 22, there is an increase in the renal nuclear fraction of MR 23.

The stimulatory effect that leptin might have on renal MR expression could be an additional way that leptin has to promote aldosterone actions. Further studies are needed to evaluate the effect of leptin replenishment on MR expression in these mouse models of leptin deficiency. Nevertheless, our data support the relationship between fat, aldosterone, and CVD; improve the understanding; and open new possibilities for the management of obesity‐related disease burden.

## Conflict of interest

The authors declare no conflict of interest.

## Author contributions

BT conceived the study, performed the experiments, analyzed data, and wrote the manuscript. SB analyzed and interpreted data and wrote the manuscript. CW performed the experiments. CC performed some experiments. FG conceived the study and participated to data interpretation. BD conceived and supervised the study and wrote the manuscript. All authors read and edited the manuscript.
